# Optimization of the QuEChERS-Based Analytical Method for Investigation of 11 Mycotoxin Residues in Feed Ingredients and Compound Feeds

**DOI:** 10.3390/toxins13110767

**Published:** 2021-10-29

**Authors:** Hyungju Seo, Sunyeong Jang, Hyeongwook Jo, Haejin Kim, Seunghwa Lee, Hyejeong Yun, Minhee Jeong, Joonkwan Moon, Taewoong Na, Hyunjeong Cho

**Affiliations:** 1 Experiment Research Institute, National Agricultural Products Quality Management Service, 141, Yongjeon-ro, Gimcheon-si 39660, Korea; hlhl103@naver.com (H.S.); wts1424@naver.com (S.J.); asarela00@korea.kr (H.K.); shlee96@korea.kr (S.L.); yhj1217@korea.kr (H.Y.); miniya33@korea.kr (M.J.); 2 Hansalim Agro-Food Analysis Center, Hankyong National University Industry Academic Cooperation Foundation, Suwon 16500, Korea; hyeongwook.jo@hknu.ac.kr (H.J.); jkmoon264@gmail.com (J.M.)

**Keywords:** mycotoxin, feed, investigation, QuEChERS, LC–MS/MS

## Abstract

Mycotoxins are toxic substances naturally produced by various fungi, and these compounds not only inflict economic damage, but also pose risks to human and animal health. The goal of the present study was to optimize the QuEChERS-based extraction and liquid chromatography–tandem mass spectrometry (LC–MS/MS) method for the analysis of 11 mycotoxins, such as aflatoxins (AFs), ochratoxin A (OTA), fumonisins (FBs), T-2 toxin, HT-2 toxin, zearalenone (ZEN), and deoxynivalenol (DON), commonly found in feed. The QuEChERS method, characterized by being “quick, easy, cheap, effective, rugged, and safe”, has become one of the most common extractions and clean-up procedures for mycotoxin analyses in food. Therefore, in this experiment, an optimal method for the analysis of 11 mycotoxins in feed was established by modifying the general QuEChERS method. In this process, it was confirmed that even if feed samples of different weights were extracted, the quantitative value of mycotoxins in the feed was not affected. To reduce matrix effects, 13C-labeled compounds and deuterium were used as internal standards. This optimized method was then applied in the determination of 11 mycotoxins in 736 feed ingredients and compound feeds obtained from South Korea. The results showed that the occurrence rates of FBs, ZEN, and DON were 59.4%, 38.0%, and 32.1%, respectively, and OTA, AFs, and T-2 toxin and HT-2 toxin were found in fewer than 1% of the 736 feeds. The mean concentration ranges of FBs, ZEN, and DON were 757–2387, 44–4552, and 248–9680 μg/kg, respectively. Among the samples in which DON and ZEN were detected, 10 and 12 samples exceeded the management recommendation standards presented by the Ministry of Agriculture, Food and Rural Affairs (MAFRA). However, when the detected concentrations of DON and ZEN were compared with guideline levels in foreign countries, such as the US, Japan, China, and the EU, the number of positive samples changed. In addition, the co-occurrence of mycotoxins in the feed was analyzed, and the results showed that 43.8% of the samples were contaminated with two or three mycotoxins, among which the co-occurrence rate of FBs, ZEN, and DON was the highest. In conclusion, these results suggest the need for stricter management standards for FBs, DON, and ZEN in South Korea, and emphasize the importance of the continuous monitoring of feeds.

## 1. Introduction

**Figure 1:**
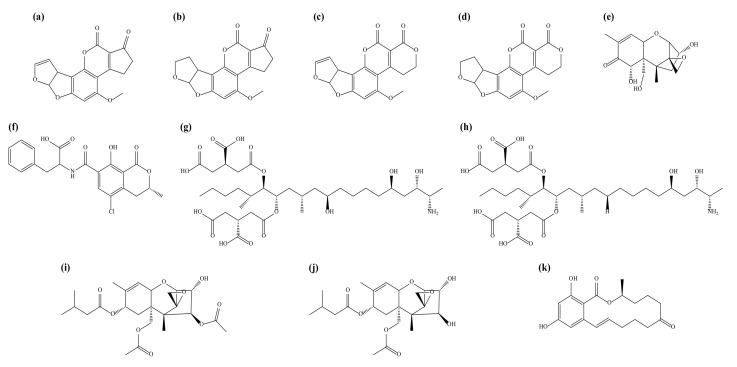
Chemical structures of the 11 mycotoxins: (**a**) aflatoxin B_1_; (**b**) aflatoxin B_2_; (**c**) aflatoxin G_1_; (**d**) aflatoxin G_2_; (**e**) deoxynivalenol; (**f**) ochratoxin A; (**g**) fumonisin B_1_; (**h**) fumonisin B_2_; (**i**) T-2 toxin; (**j**) HT-2 toxin; (**k**) zearalenone.

Mycotoxins are toxic secondary metabolites formed by fungi in genera such as *Alternaria*, *Aspergillus*, *Fusarium*, and *Penicillium* [1]. Currently, more than 500 types of mycotoxins have been identified worldwide [2]. Among the various mycotoxins, the major ones are aflatoxins (AFs), formed by *A*. *flavus* and *A*. *parasticus*; ochratoxins (OTs), produced by *A*. *ochraceus*, *A*. *carbonarius*, and *P*. *verrucosum*; trichothecenes, including type A (T-2 and HT-2 toxins) and type B (fumonisins (FBs), zearalenone (ZEN), and deoxynivalenol (DON)), mainly formed by *Fusarium* species (Figure 1); and ergot alkaloids, produced by *Claviceps* [3]. The occurrence of mycotoxins depends on factors such as the species of fungi and the host plant species. It is also influenced by climate conditions, including temperature, moisture, and relative humidity before/after harvest and between distribution and storage [4,5]. Humans and animals can be affected by ingesting food and feed contaminated with mycotoxins, or indirect mycotoxin transmission can occur when humans ingest products such as milk, eggs, and meat from livestock that has eaten feed contaminated with mycotoxins [6,7,8,9,10,11]. These toxic compounds pose health risks such as carcinogenic, teratogenic, mutagenic, nephrotoxic, and hepatotoxic effects in humans and animals, and they also have the potential to cause enormous economic losses in agriculture [12,13]. For example, FBs can induce sphingolipid metabolism disorders and activate ER stress to cause gastrointestinal damage; DON causes acute/temporary nausea, vomiting, diarrhea, abdominal pain, headache, dizziness, and fever in animals and humans; and ZEN causes hormonal imbalances in the body, which can lead to numerous diseases of the reproductive system such as prostate, ovarian, cervical, or breast cancers [14,15,16]. In particular, the International Agency for Research on Cancer (IARC) has classified DON, T-2 toxin, HT-2 toxin, and ZEN as possible human carcinogens (Group 3); FBs and ochratoxin A (OTA) as human carcinogens (Group 2B); and AF as a human carcinogen (Group 1) [17]. These toxic substances are chemically stable and do not degrade easily at high temperatures, and a single fungal species can produce various types of mycotoxins [18,19]. For these reasons, many countries have established and strictly managed maximum residue limits (MRLs) of major mycotoxins in food and feed [20]. In general, there are several important factors to consider when extracting various mycotoxins from feed. First, since each mycotoxin has specific physical and chemical properties, a solvent suitable for each mycotoxin must be selected for its extraction [18,21]. For example, FBs are hydrophilic mycotoxins and thus soluble in polar solvents, and AFs, which are hydrophobic mycotoxins, are soluble in non-polar solvents [18]. Second, the sampling and homogenization processes play important roles in determining the identity and amount of mycotoxins. This is because mycotoxin-producing fungi do not grow uniformly on the substrate, and contamination with natural specimens is not homogeneous [22]. Therefore, the sampling procedure and the homogenization process in the preparation stage before sample extraction can significantly affect the measurement of mycotoxins [23]. For this reason, the Ministry of Agriculture, Food and Rural Affairs (MAFRA) recommends passing the pulverized feed through a sieve of 20 mesh (850 μm), and the weight of the feed used for pretreatment should be set to 25 g [24]. The third factor is that feed is a complex matrix because various materials are included, and any interfering substances that remain after extraction can affect instrument analysis; hence, matrix effects must be reduced through a clean-up process [18,22,25]. 

For accurate qualitative and quantitative analyses that account for these physicochemical properties related to mycotoxins, molecular biology methods, such as enzyme-linked immunosorbent assay (ELISA), and instrument methods, including thin-layer chromatography (TLC), gas chromatography (GC), liquid chromatography (LC), and liquid chromatography–tandem mass spectrometry (LC–MS/MS), have been proposed for the analysis of mycotoxins [19]. Among them, LC–MS/MS is used for comprehensive mycotoxin analysis due to its high selectivity, high sensitivity, and ability to analyze many mycotoxins in a short time. However, quantitative analysis with LC–MS/MS can be influenced by matrix effects when an electrospray ionization source (ESI) is used [26]. The matrix effect is a change in the ionization efficiency of a compound due to the presence of a substance that elutes with the analyte of interest [27]. The matrix effect can be addressed by using certain calibration approaches, including matrix-matched calibration, the standard addition method, and through the use of internal standards [27]. In particular, internal standards are widely used in mass spectrometry not only to correct for sample preparation variations during extraction and chemical derivatization, but also to compensate for variability in signal intensity due to ion suppression caused by matrix components that may influence the efficiency of ionization [28,29,30]. 

Recently, a method of mycotoxin analysis using “quick, easy, cheap, effective, rugged, and safe” (QuEChERS)-based HPLC–MS/MS on various matrices, including food and feed, was demonstrated [21,31,32,33,34]. QuEChERS is mainly used for the analysis of pesticides, and it can be tailored to the chemical properties of various mycotoxins by using acetonitrile and water as extraction solvents [35]. In addition, the matrix effect can be minimized by using a clean-up process with a material such as primary secondary amine (PSA) or octadecyl-modified silica (C18) [36]. In recent years, several methods have been presented for mycotoxin analysis using LC–MS/MS in which quantification was attempted using a matrix-matched calibration curve [32,37,38]. This correction also helps to reduce matrix effects but the required preparation can be time- and labor-intensive, as each matrix requires a matrix-matched calibration curve, which is cumbersome when studying multiple matrices. It is also difficult to find a feed that does not contain mycotoxins to use as a matrix [39]. In addition, in previous studies in which mycotoxin analysis was performed using QuEChERS-based LC–MS/MS, there was variation in the amount of feed used for extraction, from 1 to 10 g, due to differences in the analysis target; however, few studies have provided evidence indicating the sample weight that is actually required during extraction [26,38,40,41]. 

Therefore, in this study, the samples analyzed were divided into 5 and 25 g amounts during the weighing process to ensure the homogeneity of the sample, and the amount of the detected toxin was compared to determine whether there was a difference. In addition, in order to reduce the influence of matrix effects on quantitation, deuterium- and 13C-labeled compounds were added as internal standards in the assay optimization. Finally, we applied this method in the analysis of 736 feed ingredients and compound feeds.

## 2. Results and Discussion

### 2.1. Optimization of Feed Sample Homogeneity during Sample Preparation

**Figure 2:**
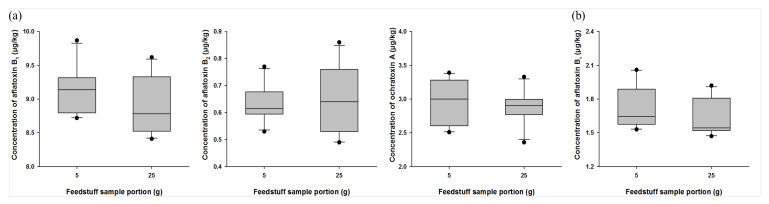
Homogeneity optimization of feed samples: (**a**) mix feed ingredient; (**b**) grain feed ingredient during sample preparation. Optimization was conducted using feed samples containing aflatoxins B_1_ and B_2_ and ochratoxin A. All samples were prepared from 1 kg of homogenized feed sample to final weights of 5 or 25 g, and this preparation was performed 10 times for each sample (*n* = 10).

Because mycotoxins present in feed are distributed in a high concentration in a local range, it is very important to select the minimum possible sample amount to be used in sample preparation while maintaining the homogeneity of the collected sample [42]. MAFRA specifies that samples that have passed through a 20-mesh (850 µm) sieve should be used for the analysis of mycotoxins present in feeds [24]. In order to establish the optimal homogeneity of the feed sample, we used 1 kg samples (mix and grain feed ingredients) containing aflatoxins B_1_ and B_2_ and ochratoxin A to perform a homogeneity test after they were ground to a level of 30 mesh (600 µm). All sample preparations were conducted 10 times from 1 kg of homogenized sample for a final weight of 5 or 25 g. For the mix feed ingredients, levels of aflatoxin B_1_ were found to be 9.13 ± 0.36 and 8.90 ± 0.42 µg/kg in the 5 and 25 g samples, respectively, and the coefficient of variation(CV) values were all within 5%. Levels of aflatoxin B_2_ were 0.63 ± 0.07 and 0.65 ± 0.13 µg/kg in the 5 and 25 g samples, respectively, and CV values were 10.80% and 19.23%, respectively. Levels of ochratoxin A were confirmed to be 2.95 ± 0.34 and 2.87 ± 0.27 µg/kg in the 5 and 25 g samples, respectively, and CV values were 11.35% and 9.37%, respectively (Figure 2a). Additionally, for grain feed ingredients, levels of aflatoxin B_1_ were verified to be 1.72 ± 0.19 and 1.63 ± 0.16 µg/kg in the 5 and 25 g samples, respectively, and CV values were 11.30% and 9.75%, respectively (Figure 2b). We observed that the recovery and CV values of both 5 and 25 g samples that were analyzed were in accordance with the international standards of AOAC and CODEX [43,44]. These results indicated that efficient analysis was possible when using sample amounts as low as 5 g during sample preparation.

### 2.2. Occurrence of 11 Mycotoxins in Feed Ingredients and Compound Feeds

The occurrence rates of 11 mycotoxins in 736 feed samples (180 feed ingredients and 556 compound feeds) were determined. In summary, feed ingredients and compound feeds contained mostly FBs, followed by ZEN and DON in terms of mycotoxins. The levels of each mycotoxin found in all feed ingredients and compound feeds are summarized in Table 1.

#### 2.2.1. Aflatoxins B_1_, B_2_, G_1_, and G_2_

Of the total of 736 samples, AFs were detected in 1 sample, which was a vegetable meal feed. Other than this sample, feed ingredients and compound feeds were not found to be contaminated with AFs. According to the MAFRA guidance from South Korea, MRLs of aflatoxins B_1_, B_2_, G_1_, and G_2_ in feeds are defined for the sum of these toxins: MAFRA MRLs of AFs are 50 µg/kg in feed ingredients and 10 µg/kg in compound feeds [24]. In this study, only one positive sample was found to be contaminated with AF, which was present at a level of 159 µg/kg in vegetable meal feed and exceeded the MAFRA MRL of 50 µg/kg in feed ingredients. In addition, the contamination level of aflatoxin B_1_ in vegetable meal feed was 108 µg/kg, which is higher than the regulatory limits of 10 µg/kg in China and Japan and 20 µg/kg in the EU, as set by the European Commission (EC) [45,46,47].

Many studies on AF contamination of feed have been performed worldwide. In South Korea, Kim et al. (2017) analyzed 507 grain feed samples and found AF contamination in 1.0% of feed samples. The contamination levels ranged from 1 to 12 µg/kg [48]. Additionally, no feed samples showed higher contamination levels than the MAFRA MRL of 50 µg/kg [24]. In another study in South Korea, Kim et al. (2017) monitored 1014 feed samples from 2015 to 2016 and found that AF contamination levels ranged from 2 to 163 µg/kg [49]. One animal protein sample was found to be contaminated with an AF level of 163 µg/kg, which exceeded the MAFRA MRL of 50 µg/kg in feed ingredients [24]. In China, Ma et al. (2018) monitored 1569 feed samples from 2016 to 2017 and found aflatoxin B_1_ contamination in 83.3%, and levels ranged from 2 to 68 µg/kg [50]. In a recent similar study, Zhao et al. (2021) measured aflatoxin B_1_ levels in 3507 feed samples collected from 2018 to 2020, and the contamination range was 1–221 µg/kg [51]. Moreover, aflatoxin B_1_ contamination in 9 feed ingredient samples and 63 compound feed samples exceeded the regulatory limits in China [45,50,51]. In Japan, Nomura et al. (2017) measured aflatoxin B_1_ in 1233 feed samples from 2010 to 2015 and found that 46.0% of the total feed samples were contaminated with aflatoxin B_1_, with contamination levels ranging from 1 to 24 µg/kg [52]. Monitoring results showed that contamination in five feed samples exceeded the regulatory limit of 20 μg/kg in Japan [46]. Additionally, Uegaki et al. (2018) performed an analysis to determine the levels of aflatoxin B_1_ in 214 feed samples collected from livestock farming establishments in Japan [53]. The contamination range exceeded the Japanese regulatory limit of 1000 μg/kg in one grain feed, and other feeds showed a range of 1–22 μg/kg [46]. In Poland, Grajewski et al. (2012) monitored 1255 feed samples for the occurrence of AFs, and 7.0% of feed samples were found to be contaminated with AFs. The contamination range was 0–1 µg/kg [54]. In Turkey, Bilal et al. (2014) surveyed 106 feed samples to estimate aflatoxin B_1_ contamination levels, and the contamination range was 1–11 µg/kg [55]. In Spain, Manzanares et al. (2019) performed a similar study and reported that 3.1% of 288 pig feed samples were contaminated with aflatoxin B_1_ at levels ranging from 0 to 3 µg/kg [56]. None of the feed samples studied in European countries were found to exceed the EC regulatory limits for feed ingredients and compound feeds (20 and 10 μg/kg, respectively) [47,54,55,56].

The moderate climatic conditions of Europe make it difficult for aflatoxigenic *Aspergillus* spp. to infiltrate crops. On the other hand, hot and humid climatic conditions in Asia increase the likelihood of exposure to aflatoxin-causing fungi in maize and other crops, and a similar trend was observed in this study. A study by Dorninger et al. also reported that hot and humid weather conditions affect AF contamination [57].

#### 2.2.2. Deoxynivalenol

DON was detected in one-third (236) of the total 736 samples. DON was most frequently detected in pig feed (79.2%), and levels ranged from 53 to 1274 µg/kg. The rates of DON contamination in feed ingredients were 16.6%, 28.0%, 17.5%, and 7.9% in grain, bran, vegetable meal, and mix feeds, respectively. By contrast, animal protein and mineral feeds were not contaminated with DON. In addition, over half of the collected compound feed samples showed DON contamination. The DON contamination rates in compound feeds were 73.7%, 79.2%, 50.0%, 64.3%, and 26.8% in poultry, pig, dairy, cattle, and pet feeds, respectively. The occurrence of DON in 736 feed samples levels ranged from 51 to 19,529 µg/kg. The maximum level was 19,529 µg/kg in pet feed, followed by 18,785 µg/kg in grain feed. The median level was 9680 µg/kg in grain feed, followed by 2008 µg/kg in dairy feed. 

According to the MAFRA guidance from South Korea, the recommended limits of DON are 5000 µg/kg in feed ingredients and, among compound feeds, 5000 µg/kg in poultry and pet feeds, 2000 µg/kg in dairy and cattle feeds, and 900 µg/kg in pig feed [24]. A total of 10 positive samples contained DON levels above the limits recommended by MAFRA; the 10 samples comprised 1 feed ingredient (grain feed) and 9 compound feeds, including 2 pig feeds, 1 dairy feed, 5 cattle feeds, and 1 pet feed. Notably, in a total of five cattle feed samples, which account for 11.9% of all cattle feed samples, the contamination range of DON was 2075–13,181 µg/kg, which exceeded the MAFRA recommended limit of 2000 µg/kg. 

The measured values were compared with regulatory or guidance limits in other countries, and the results showed that one grain feed sample was higher than the regulatory limit of 5000 µg/kg established by China and the EC guidance limit of 8000 µg/kg [45,58]. Four grain feed samples exceeded the regulatory limit of 1000 µg/kg in Japan [46]. Furthermore, two pig feed samples exceeded the regulatory limit of 1000 µg/kg in China and Japan and the EC guidance limit of 900 µg/kg [45,46]. In addition, three cattle feed samples exceeded the regulatory limits of 3000 µg/kg in China, 4000 µg/kg in Japan, and the EC guidance limit of 5000 µg/kg [45,46,58]. By contrast, three poultry and three dairy feed samples that did not exceed the MARFA recommended limit of 5000 µg/kg had higher contamination levels than the regulatory limit of 1000 µg/kg in Japan [46].

Many studies have been conducted worldwide on the level of DON contamination in feeds. In a previous study in South Korea, Park et al. (2018) monitored 653 feed samples collected from 2009 to 2016 and found that 79.7% had DON contamination, with ranging from 1 to 8480 µg/kg [59]. The maximum level of DON was identified in bran feed and did not exceed the MAFRA recommended limit of 10,000 µg/kg [24]. In another previous study, the contamination level of DON tended to be lower than the levels detected in this study. In China, Wu et al. (2016) detected DON in 93.9% of 560 feed samples that were collected from 2013 to 2015. The contamination levels ranged from 349 to 4403 µg/kg [60]. Ma et al. (2018) monitored 1569 feed samples from 2016 to 2017 and found DON contamination in 74.5%, with levels ranging from 450 to 12,633 µg/kg [50]. In a recent report, Zhao et al. (2021) analyzed 3507 feed samples collected from 2018 to 2020 and found DON contamination in 96.4%, with contamination levels ranging from 458 to 9186 µg/kg [51]. In Japan, Uegaki et al. (2018) analyzed 214 feed samples. The DON contamination rate in feeds was 64.0%, and 11 feed samples exceeded the regulatory limit of 1000 μg/kg in Japan [46,53]. In Portugal, Almeida et al. (2011) analyzed 277 pig feed samples and confirmed that 16.9% of total feeds were contaminated with DON in a range of 100–864 µg/kg [61]. Additionally, no pig feed samples were found to have contamination levels higher than the EC guidance limit of 900 µg/kg [58]. In Poland, Grajewski et al. (2012) monitored 1255 feed samples collected from 2006 to 2009 and found DON contamination in 88.0% of feeds. The contamination levels ranged from 409 to 7356 µg/kg [54]. The concentration of DON did not exceed the EC guidance limit of 8000 µg/kg for grain feeds [58]. In Turkey, Bilal et al. (2014) measured the levels of DON in 106 feed samples. The contamination rate was 43.4%, and levels ranged from 37 to 4770 µg/kg [55]. The highest contamination level was identified in corn feed, but it did not exceed the EC guidance limit of 8000 µg/kg [58]. 

High rainfall and warm temperatures during the growing season of wheat or corn have been shown to increase the occurrence of DON. High rainfall during the growing season can promote contamination with *Fusarium* spp. in corn or wheat, while continued rainfall during harvest provides adequate moisture to sustain fungal growth and mycotoxin production within the grain. In particular, higher DON concentrations occurred in East Asia with heavy rainfall in August and September compared to those in European countries [57]. 

#### 2.2.3. Fumonisins B_1_ and B_2_

FBs were detected in more than half (437) of the total of 736 samples. FBs were most frequently detected in cattle feed (100.0%), and the levels ranged from 66 to 23,422 µg/kg. The FB contamination rates in feed ingredients were 33.3%, 64.0%, 40.0%, and 11.1% in grain, bran, vegetable meal, and mix feeds, respectively. By contrast, animal protein and mineral feeds were not contaminated with FBs. Over half of the collected compound feed samples showed FB contamination with 89.5%, 90.6%, 95.0%, 100.0%, and 62.3% in poultry, pig, dairy, cattle, and pet feeds, respectively. These results showed that the occurrence of FBs in feed ingredients and compound feeds was similar to that of DON. The levels of FBs in 736 feed samples ranged from 43 to 23,422 µg/kg. The maximum level was 23,422 µg/kg in cattle feed, followed by 10,486 µg/kg in bran feed. The median level was 2387 µg/kg in bran feed, followed by 2360 µg/kg in cattle feed. 

According to the MAFRA guidance from South Korea, the recommended limits of fumonisin B_1_ and B_2_ in feeds are defined for the sum of these toxins. The MAFRA recommended limit of FBs in feed ingredients is 60,000 µg/kg; in compound feeds, the recommended limits are 50,000 µg/kg in dairy and cattle feeds, 20,000 µg/kg in poultry feed, and 5000 µg/kg in pig and pet feeds [24]. In contrast to the 10 positive samples exceeding MAFRA-recommended limits of DON, no FB-positive samples that exceeded the MAFRA recommended limits in feed ingredients and compound feeds were identified. Additionally, none of the analyzed feed samples were found to have FB contamination levels exceeding the EC guidance limit [58]. 

In a previous report in South Korea, Kim et al. (2014) analyzed 180 feed samples for the occurrence of FBs, and 91.7% were found to be contaminated with FBs [62]. The contamination range was 37–12,823 µg/kg, and the contamination level of FBs in one pig feed sample exceeded the MAFRA recommended limit of 5000 µg/kg [24]. Additionally, Park et al. (2017) monitored 535 feed samples collected from 2011 to 2016 to estimate FB contamination levels [63]. The contamination range was 15–15,098 µg/kg, and seven pig feed samples exceeded the MAFRA-recommended limit of 5000 μg/kg [24]. In Poland, Grajewski et al. (2012) examined FB contamination levels in 1255 feed samples and showed that the contamination rate and level were 78.0% and 10–9409 µg/kg, respectively [54]. The maximum level of FBs was identified in grain feeds, which did not exceed the EC guidance limit of 60,000 µg/kg [58]. In Spain, Manzanares et al. (2019) analyzed 288 pig feed samples, of which 79.8% were contaminated with FBs [56] in the range of 4–3959 µg/kg. No feed samples were found to exceed the EC guidance limit of 5000 μg/kg [58]. 

FB contamination is closely related to high temperatures and high precipitation during the growing season of grains. Thus, regions with warm, dry climates such as America and East Asia have particularly high levels of contamination. Consequently, previous studies have shown that FB contamination levels in South Korea, China, and Japan are higher than those in European countries [57]. 

#### 2.2.4. Ochratoxin A

Of the total 736 samples, OTA was detected in three samples, which were bran, poultry, and pet feeds. Except for the above three feed samples, OTA was not found in the other feed ingredients and compound feeds that were examined. According to the MAFRA guidance from South Korea, the MRLs of OTA are 250 µg/kg in feed ingredients and 200 µg/kg in compound feeds [24]. In this study, we found that bran, poultry, and pet feeds were contaminated with OTA levels of 61, 18, and 11 μg/kg, respectively, which did not exceed the MAFRA MRL. 

In a previous report in South Korea, Kim et al. (2017) monitored 507 grain feed samples and identified 1 that was contaminated with OTA at a level of 0.5 µg/kg [48]. A similar study was conducted by Kim et al. (2017) to determine the levels of OTA in 1014 feed samples collected from 2015 to 2016 in South Korea [49], where the contamination range was 2–45 µg/kg, which did not exceed the MAFRA MRL of 200 µg/kg [24]. In Portugal, Almeida et al. (2011) examined 277 pig feed samples and confirmed that 7.6% of total feeds were contaminated with OTA in a range from 2 to 7 µg/kg [61]. In Poland, Grajewski et al. (2012) performed a similar study and reported that 30.8% of 1255 feed samples were contaminated with OTA, with contamination levels ranging from 1 to 760 µg/kg [54]. Among these 1255 feed samples, 2 feed ingredients and 4 pig feed samples were found to exceed the EC guidance limits of 250 and 50 μg/kg, respectively [58]. Although the levels of OTA occurrence in South Korea are low compared to other European countries, continuous management is required.

#### 2.2.5. T-2 and HT-2 Toxins

Of the total of 736 samples, T-2 and HT-2 toxins were detected in 1 sample, which was a vegetable meal feed. The remaining samples were not found to be contaminated with T-2 and HT-2 toxins. According to the MAFRA guidance from South Korea, the recommended limits for the overall combined level of T-2 and HT-2 toxins in are defined individually for their use as feed ingredient of in compound feeds; the MAFRA recommended limits of T-2 and HT-2 toxins are 500 µg/kg for feed ingredients and 250 µg/kg for compound feeds [24]. In this study, only vegetable meal feed was found to be contaminated with T-2 and HT-2 toxins; the T-2 and HT-2 toxin level was 40 µg/kg, which did not exceed the MAFRA recommended limit or the EC guidance limit of 500 µg/kg in feed ingredients [24,58]. 

In a previous report in South Korea, Ok et al. (2013) carried out an analysis on T-2 and HT-2 toxin levels in 214 grain feed samples obtained from grocery markets, and found a contamination range of 6–207 µg/kg [64]. In a similar study, Kim et al. (2017) analyzed 507 grain feed samples, and 2.0% were found to be contaminated with T-2 and HT-2 toxins. The contamination range was 4–14 µg/kg [48]. In Poland, Grajewski et al. (2012) monitored T-2 and HT-2 toxins in 1255 feed samples from 2006 to 2009, and found that 26.0% of the total feed samples were contaminated with T-2 and HT-2 toxins, and the contamination levels ranged from 1 to 289 µg/kg [54]. In Spain, Manzanares et al. (2019) analyzed 288 pig feed samples and found T-2 and HT-2 toxin contamination in 96.4%. The contamination levels ranged from 458 to 9186 µg/kg [56]. Contamination levels of T-2 and HT-2 toxins in our study were found to be lower than those of previous reports.

#### 2.2.6. Zearalenone

ZEN was detected in about 38.0% (280) of the total of 736 feed samples. ZEN was most frequently detected in dairy feed (90.0%), and levels ranged from 24 to 2212 µg/kg. The ZEN contamination rates in feed ingredients were 33.3%, 48.0%, 3.7%, 22.5%, and 12.7% in grain, bran, animal protein, vegetable meal, and mix feeds, respectively. By contrast, the mineral feed was not contaminated with ZEN. In addition, ZEN contamination was detected in over half of the collected compound feed samples. The contamination rates of ZEN in compound feeds were 65.8%, 67.9%, 90.0%, 88.1%, and 32.3% in poultry, pig, dairy, cattle, and pet feeds, respectively. The ZEN levels ranged from 13 to 18,645 µg/kg among the 736 feed samples. The maximum level was 18,645 µg/kg in cattle feed, followed by 18,113 µg/kg in grain feed. The median level was 4552 µg/kg in grain feed, followed by 523 µg/kg in bran feed. 

According to the MAFRA guidance from South Korea, the recommended limits of ZEN are 3000 µg/kg in feed ingredients, and among compound feeds, 500 µg/kg in poultry, dairy, and pet feeds; 100 µg/kg in pig feed; and 1000 µg/kg in pet feed [24]. A total of 12 positive samples were contaminated with ZEN levels above the MAFRA recommended limits; the 12 samples comprised 1 feed ingredient (grain feed) and 11 compound feeds, including 1 poultry feed, 2 pig feeds, 2 dairy feeds, 5 cattle feeds, and 1 pet feed. Notably, in a total of five cattle feed samples, which accounted for 11.9% of all cattle feed samples, the contamination range of ZEN was 21–18,645 µg/kg, which exceeded the MAFRA recommended limit of 500 µg/kg. 

The measured values were compared with regulatory or guidance limits in other countries, and the results showed that one grain feed sample was higher than the EC guidance limit of 2000 µg/kg [58]. Five grain and one poultry feed sample exceeded the regulatory limit of 1000 µg/kg in China and Japan [45,46]. In addition, two pig feed samples exceeded the EC guidance limit of 100 µg/kg [58]. Furthermore, two dairy and five cattle feed samples had higher contamination levels than the Chinese regulatory limit and the EC guidance limit of 500 µg/kg, respectively [45,58]. 

Many studies on ZEN contamination in feeds have been performed worldwide. In a previous study in South Korea, Kim et al. (2014) analyzed 180 feed samples and found ZEN contamination in 62.8% of all feeds [62]. The contamination levels ranged from 8 to 413 µg/kg, and several pig feed samples showed higher contamination levels than the MAFRA-recommended limit of 100 µg/kg [24]. In a similar study, Kim et al. (2017) conducted an analysis to determine the levels of ZEN in 507 grain feed samples [48]. The contamination range was 1–313 µg/kg, which did not exceed the MAFRA-recommended limit of 3000 µg/kg [24]. Chang et al. (2017) confirmed that 86.7% of 653 feed samples collected from 2009 to 2016 were contaminated with ZEN, and the contamination levels ranged from 1 to 1330 µg/kg [65]. Two cattle and two pig feed samples exceeded the MAFRA-recommended limits of 500 and 100 μg/kg, respectively [24]. In China, Ma et al. (2018) monitored ZEN levels in 1569 feed samples collected from 2016 to 2017. They found ZEN contamination in 88.0% of samples, and the contamination range was 2–1363 µg/kg [50]. Zhao et al. (2021) monitored 3507 feed samples from 2018 to 2020 and found ZEN contamination in 96.9%, and levels ranged from 31 to 1599 µg/kg [51]. Moreover, ZEN contamination in 71 feed ingredients and 37 compound feeds exceeded the Chinese regulatory limits of 500 and 250 μg/kg, respectively [45,50,51]. In Japan, Uegaki et al. (2018) measured the levels of ZEN in 214 feed samples. The contamination rate was 49.0%, and levels ranged from 46 to 1200 µg/kg [53]. The highest contamination was identified in grass feed samples, two of which exceeded the regulatory limit of 1000 µg/kg in Japan [46]. In Portugal, Almeida et al. (2011) monitored ZEN in 404 pig feed samples and found that 19.1% of the total feed samples were contaminated with ZEN, and the contamination levels ranged from 5 to 73 µg/kg [61]. In Turkey, Bilal et al. (2014) examined 106 feed samples and confirmed that 43.4% of total feeds were contaminated with ZEN in a range from 3 to 97 µg/kg [55]. Additionally, none of the studied feed samples had levels of ZEN contamination that exceeded the EC guidance limits [58]. In Spain, Manzanares et al. (2019) analyzed 228 pig feed samples and confirmed that 7.0% of total feeds were contaminated with ZEN in a range from 101 to 956 µg/kg [56]. Additionally, six pig feed samples showed higher contamination levels than the EC guidance limit of 100 µg/kg [58]. 

The increase in the occurrence of ZEN, one of the well-known toxins produced by *Fusarium* spp. in addition to DON, was influenced by high rainfall and warm temperatures during the growing season of wheat or corn. High rainfall during the growing season can promote contamination with *Fusarium* spp. in corn or wheat, while continued rainfall during harvest can provide adequate moisture for sustained fungal growth and mycotoxin production within the grain. In particular, higher ZEN concentrations occurred in East Asia with heavy rainfall in August and September compared to values reported in previous studies in European countries [57].

### 2.3. Co-Occurrence of Mycotoxins in Feed Ingredients and Compound Feeds

The co-occurrence of DON, FBs, OTA, T-2 toxin, HT-2 toxin, and ZEN in feed samples is shown in Table 2. Of the total samples, 36.5% were not contaminated with any mycotoxins, 19.7% were contaminated with a single mycotoxin, and 43.8% were simultaneously contaminated with two or more mycotoxins, and the occurrence rates were 9.6–48.0% in feed ingredients and 39.7–95.2% in compound feeds. These results are supported by previous studies showing that compound feeds are particularly susceptible to multiple contaminations because they typically contain several raw materials [8]. The co-occurrence of DON, ZEN, and FBs, which are known to be produced by the genus *Fusarium*, was detected most frequently in compound feeds in particular combinations, in the following order of decreasing prevalence: DON+FBs+ZEN (15.9–62.3%) > ZEN+FBs (5.7–45.0%) > DON+FBs (5.0–13.2%). In particular, nine samples (one grain, one pig, one dairy, five cattle, and one pet feed) were simultaneously contaminated by DON+ZEN+FBs, and DON and ZEN exceeded the limits suggested by MARFA [24]. The concentration ranges of DON and ZEN were 1274–19,529 and 143–18,645 μg/kg, respectively. Previous studies have shown that co-contamination with DON and ZEN resulted in an additive or synergistic risk or antagonistic effects in animals such as pigs, rats, and mice [57]. A study of the co-occurrence of ZEN and DON showed that the toxins may induce mitochondrial apoptosis processes in humans, as indicated in a cytotoxicity assay using a human colon cancer cell line (HCT116) [66]. In addition, in human granulocyte hematopoietic progenitor cells, DON and ZEN were reported to have an additive effect on apoptosis resulting from the stimulation of caspase-3 activity [67]. In addition, it was suggested that when simultaneously contaminated by aflatoxin b1, DON, and ZEN, mouse and rat liver cells may have a higher hepatotoxic than individual contamination [68,69]. These results suggest that the toxicity of co-contaminating mycotoxins may not be possible to predict based only on the mechanisms of the individual toxins [70]. The co-occurrence of mycotoxins is important in that mycotoxigenic fungi are capable of producing more than one mycotoxin, and are influenced by the surrounding environment and the multiple raw materials found in feeds [62,71,72]. Thus, studying the contamination of a single mycotoxin cannot provide sufficient information about the risks associated with each feed [73]. Furthermore, simultaneous contamination with mycotoxins has been shown to have greater negative effects on health and productivity than with single mycotoxins [74]. The co-occurrence of mycotoxins in feeds has been consistently reported in many countries [50,51,61,75,76]. However, current safety regulations do not consider the toxic potential of co-occurring mycotoxins, so it is necessary to establish strict management standards for the co-occurrence of mycotoxins, and to continuously monitor them in feeds.

## 3. Conclusions

In this study, the QuEChERS-based LC–MS/MS method was optimized to simultaneously quantify 11 mycotoxins contained in feeds, whereby deuterium- and 13C-labeled compounds were used as internal standards. In addition, it was confirmed that there was no significant difference in the amount of mycotoxin detected when the weight of corn feed differed in the pretreatment stage. This optimized method was applied to 736 samples of feed ingredients and compound feeds commonly consumed in South Korea. The results showed that of the 11 examined mycotoxins, DON, FBs, and ZEN were most frequently detected in feeds. Among them, DON and ZEN were found to exceed the guidance limits set by MAFRA in 10 and 12 samples, respectively. Most of the positive samples were identified in compound feeds. Therefore, it is necessary to more strictly manage the regulation and guideline levels of contamination with mycotoxins such as DON, FBs, and ZEN in compound feeds in South Korea. In addition, these results suggested the need for continuous monitoring of mycotoxins in feed ingredients and compound feeds. 

## 4. Materials and Methods

### 4.1. Chemical and Reagents

All mycotoxin standards and internal standards (ISs) were provided by Sigma-Aldrich (St. Louis, MO, USA) and Cfm Oskar Tropitzsch GmbH (Marktredwitz, Germany). Acetonitrile (ACN), methanol (MeOH), and water were purchased from Merck (Darmstadt, Germany). All solvents used in the analysis were LC–MS grade. Purified water was obtained using a Milli-Q system (Millipore, Bedford, MA, USA). Ammonium formate was obtained from Sigma-Aldrich (St. Louis, MO, USA). Formic acid was supplied by Fisher Scientific (Pittsburgh, PA, USA). Bondesil primary secondary amine (PSA) and octadecylsilane (C18) were provided by Biotage (Uppsala, Sweden). QuEChERS salt mixture containing 4 g of MgSO_4_ and 1 g of NaCl was purchased from BEKOlut GmbH & Co. KG (Hauptstuhl, Germany).

### 4.2. Samples

For mycotoxin monitoring in feeds commonly used in South Korea, a total of 736 feed samples (180 feed ingredients and 556 compound feeds) were randomly obtained from feed factories and markets in various locations in South Korea. All feed samples were ground using an SM 300 cutting mill (Retsch, Germany) to a particle size <4 mm and stored in the freezer at −20 °C.

### 4.3. Preparation of Standard Solutions

Individual stock solutions of mycotoxins and IS were dissolved in ACN or MeOH. Working standard solutions were prepared with ACN for the calibration curve, constructed at each concentration based on MAFRA from South Korea [24]. All solutions were placed in amber glass vials and stored in the freezer at −20 °C.

### 4.4. Calibration Curve

Internal standard calibration curves were plotted with five points for each mycotoxin based on MAFRA from South Korea. The calibration ranges of 11 mycotoxins were evaluated as 0.5–25 ng/mL for aflatoxins B_1_, B_2_, G_1_, and G_2_; 10–500 ng/mL for deoxynivalenol, fumonisins B_1_ and B_2_, T-2 toxin, and HT-2 toxin; 2–100 ng/mL for ochratoxin A; and 2.5–125 ng/mL for zearalenone [32]. An internal standard (IS) labeled with isotope or deuterium was used for quantification. The analysis was performed under established LC–MS/MS conditions, and the calibration curve was prepared in the form of y = ax + b (y: peak area; x: concentration).

### 4.5. Sample Preparation Using Optimized QuEChERS Method

**Figure 3:**
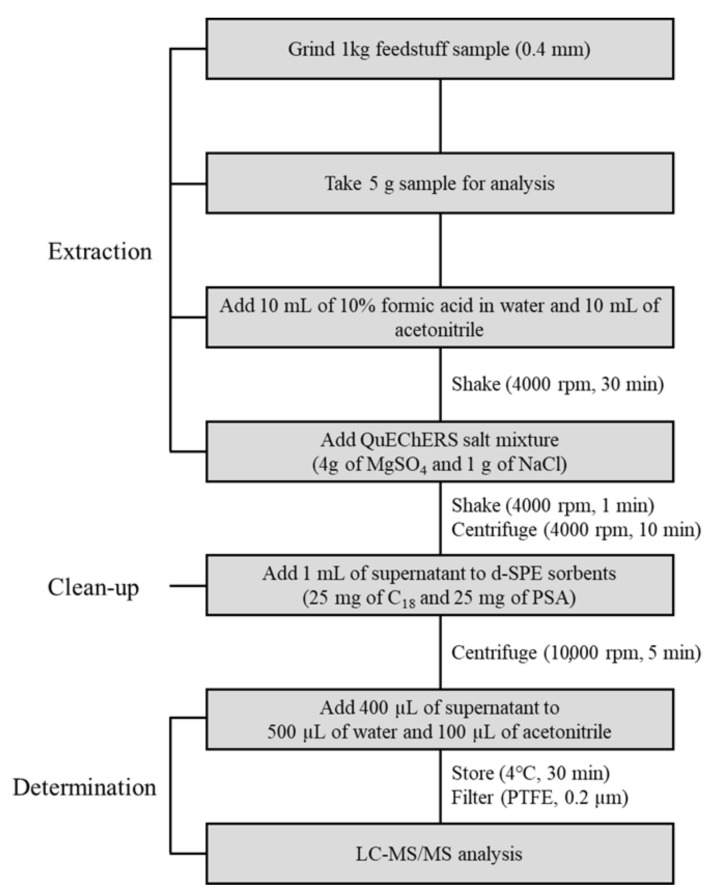
Analytical flow of the optimized QuEChERS method for the analysis of mycotoxins in feed samples.

Prior to their extraction, 1 kg feed samples with representative properties were ground and homogenized using a cutting mill. About 5 g of each ground sample was extracted with 10 mL of acetonitrile and 10 mL of water with 10% formic acid in a homogenizer at 4000 rpm for 30 min. After extraction, 4 g of MgSO_4_ and 1 g of NaCl were added to the samples, and solutions were shaken at 4000 rpm for 1 min. Extracted samples were centrifuged at 4000 rpm for 10 min. For sample clean up, 1 mL of the supernatant obtained from centrifugation was added to a 1.5 mL centrifuge tube containing 25 mg of C18 and 25 mg of PSA and shaken at 10,000 rpm for 5 min. After centrifugation, 400 μL aliquots of supernatant were transferred into a 1.5 mL centrifuge tube and mixed with 500 μL of distilled water and 100 μL of ACN, which was stored in the refrigerator at 4 °C for 30 min. All samples were filtered using a 0.2 μm polytetrafluoroethylene (PTFE) syringe filter and stored in plastic vials. The sample extracts were analyzed by liquid chromatography–tandem mass spectrometry (LC–MS/MS) [32]. All sample preparations were performed in triplicate. The analytical flow of the optimized QuEChERS extraction method for the analysis of mycotoxins in feed samples is shown in Figure 3.

### 4.6. LC–MS/MS Analysis

**Figure 4:**
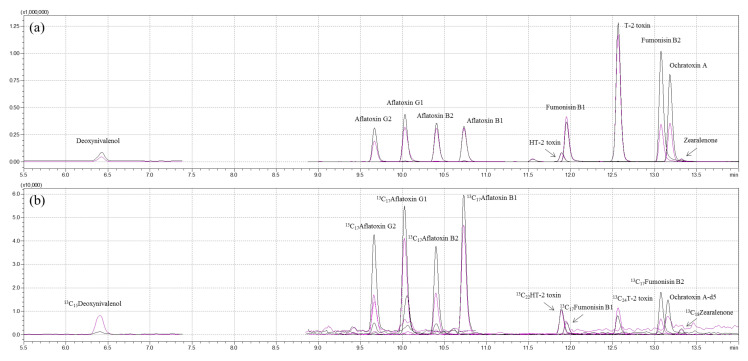
Representative multiple-reaction monitoring chromatograms of: (**a**) 11 mycotoxin standards; (**b**) internal standards.

The determination of 11 mycotoxins in feed ingredients and compound feeds was performed in accordance with a previous report with slight modifications [32]. All mycotoxins and the internal standards were analyzed on a Shimadzu LC–MS 8050 triple-quadrupole mass spectrometer equipped with a Nexera X2 ultra-high-performance liquid chromatography system (Shimadzu, Kyoto, Japan) coupled with an electrospray ionization (ESI) interface. Chromatographic separation was carried out in an Imtakt Cardenza CD-C18 UP column (150 mm × 2.0 mm, 3.0 μm, 120 Å), and the column oven temperature was maintained at 40 °C. Water with 5 mM ammonium formate and 0.1% formic acid (A) and acetonitrile with 5 mM ammonium formate and 0.1% formic acid (B) were used as mobile phases. The high-performance liquid chromatography (HPLC) separation used a linear gradient program of 5% B for 0–2.0 min, 5%–100% B for 2.0–15.0 min, 100–5% B for 15.0–15.1 min, and 5% B for 15.1–18.0 min. The total run time was 18.0 min, and the flow rate was 0.4 mL/min. In order to minimize interference between the analyte and the IS, the first feed sample was injected, and the IS mixture contained in another vial was then injected at a volume of 2 µL using the autosampler pretreatment function. The analysis was performed using electrospray ionization (ESI) in positive-ion mode at 4000 V or in negative-ion mode at −4000 V. The instrument parameters were optimized as follows: interface temperature, 300 °C; heat block temperature, 400 °C; DL temperature, 250 °C; nebulizing gas flow, 3 L/min; heating gas flow, 10 L/min; drying gas (nitrogen) flow, 10 L/min. For the quantitation and qualification of each mycotoxin, multiple-reaction monitoring (MRM) was performed. Precursor-ion- and product-ion-optimized MS/MS conditions and chromatograms for each mycotoxin are described in Table 3 and Figure 4.

### 4.7. Data Analysis

All the analytical data were processed using the Shimadzu LabSolutions LC–MS software (Shimadzu, Kyoto, Japan). The median values of experimental data were calculated using Microsoft Excel 2013 (Microsoft Co., Redmond, WA, USA). Statistical analysis of box and whisker plots was performed with SigmaPlot 12.0 (Systat Software Inc., Erkrath, Germany).

## Figures and Tables

**Table 1:** Levels of 11 mycotoxins in 736 feed ingredients and compound feeds in South Korea in 2020 (*n* = 3).

Sample		No. of Samples	No. of Detected Samples ^1^ (%)	No. of Positive Samples ^2^ (%)	Minimum (µg/kg)	Median (µg/kg)	Maximum (µg/kg)	Guidance Limit ^3^ (ppb)
Aflatoxin B_1_ + B_2_ + G_1_ + G_2_								
Feed ingredients	Grain	12	-	-	-	-	-	50
	Bran	25	-	-	-	-	-	50
	A. protein ^4^	27	-	-	-	-	-	50
	V. meal ^5^	40	1 (2.5)	1 (2.5)	159	159	159	50
	Mineral	13	-	-	-	-	-	50
	Mix	63	-	-	-	-	-	50
Compound feeds	Poultry	38	-	-	-	-	-	10
	Pig	53	-	-	-	-	-	10
	Dairy	20	-	-	-	-	-	10
	Cattle	42	-	-	-	-	-	10
	Pet	403	-	-	-	-	-	10
Deoxynivalenol								
Feed ingredients	Grain	12	2 (16.6)	1 (8.3)	575	9680	18,785	10,000
	Bran	25	7 (28.0)	-	464	1738	3478	10,000
	A. protein	27	-	-	-	-	-	10,000
	V. meal	40	7 (17.5)	-	79	821	1495	10,000
	Mineral	13	-	-	-	-	-	10,000
	Mix	63	5 (7.9)	-	54	248	542	10,000
Compound feeds	Poultry	38	28 (73.7)	-	55	512	2941	5000
	Pig	53	42 (79.2)	2 (3.8)	53	328	1274	900
	Dairy	20	10 (50.0)	1 (5.0)	138	2008	2525	2000
	Cattle	42	27 (64.3)	5 (11.9)	74	1884	13,181	2000
	Pet	403	108 (26.8)	1 (0.2)	51	472	19,529	5000
Fumonisin B_1_ + B_2_								
Feed ingredients	Grain	12	4 (33.3)	-	67	2054	7958	60,000
	Bran	25	16 (64.0)	-	60	2387	10,486	60,000
	A. protein	27	-	-	-	-	-	60,000
	V. meal	40	16 (40.0)	-	43	1064	2808	60,000
	Mineral	13	-	-	-	-	-	60,000
	Mix	63	7 (11.1)	-	195	757	1462	60,000
Compound feeds	Poultry	38	34 (89.5)	-	69	812	5985	20,000
	Pig	53	48 (90.6)	-	80	967	2059	5000
	Dairy	20	19 (95.0)	-	127	1286	3668	50,000
	Cattle	42	42 (100.0)	-	66	2360	23,422	50,000
	Pet	403	251 (62.3)	-	50	903	3397	5000
Ochratoxin A								
Feed ingredients	Grain	12	-	-	-	-	-	250
	Bran	25	1 (4.0)	-	61	61	61	250
	A. protein	27	-	-	-	-	-	250
	V. meal	40	-	-	-	-	-	250
	Mineral	13	-	-	-	-	-	250
	Mix	63	-	-	-	-	-	250
Compound feeds	Poultry	38	1 (2.6)	-	18	18	18	200
	Pig	53	-	-	-	-	-	200
	Dairy	20	-	-	-	-	-	200
	Cattle	42	-	-	-	-	-	200
	Pet	403	1 (0.2)	-	11	11	11	200
T-2 toxin, HT-2 toxin								
Feed ingredients	Grain	12	-	-	-	-	-	500
	Bran	25	-	-	-	-	-	500
	A. protein	27	-	-	-	-	-	500
	V. meal	40	1 (2.5)	-	40	40	40	500
	Mineral	13	-	-	-	-	-	500
	Mix	63	-	-	-	-	-	500
Compound feeds	Poultry	38	-	-	-	-	-	250
	Pig	53	-	-	-	-	-	250
	Dairy	20	-	-	-	-	-	250
	Cattle	42	-	-	-	-	-	250
	Pet	403	-	-	-	-	-	250
Zearalenone								
Feed ingredients	Grain	12	4 (33.3)	1 (8.3)	16	4552	18,113	3000
	Bran	25	12 (48.0)	-	15	523	1709	3000
	A. protein	27	1 (3.7)	-	18	18	18	3000
	V. meal	40	9 (22.5)	-	15	275	931	3000
	Mineral	13	-	-	-	-	-	3000
	Mix	63	8 (12.7)	-	15	61	121	3000
Compound feeds	Poultry	38	25 (65.8)	1 (2.6)	15	128	1370	500
	Pig	53	36 (67.9)	2 (3.8)	14	44	143	100
	Dairy	20	18 (90.0)	2 (10.0)	24	286	2212	500
	Cattle	42	37 (88.1)	5 (11.9)	21	368	18,645	500
	Pet	403	130 (32.3)	1 (0.2)	13	192	17,268	1000

^1^ Level of mycotoxin higher than LOQ; ^2^ level of mycotoxin higher than guidance limit; ^3^ guidance for mycotoxin levels in feed according to Ministry of Agriculture, Food and Rural Affairs (MAFRA) from South Korea; ^4^ animal protein; ^5^ vegetable meal.

**Table 2:** Co-occurrence of 11 mycotoxins in 736 feed ingredients and compound feeds.

Sample		No. of Samples	No. of Co-OccurrenceSamples (%)	DON ^1^ + FBs ^2^ (%)	DON + ZEN ^3^ (%)	FBs + OTA ^4^ (%)	FBs + T-2/HT-2 (%)	FBs + ZEN (%)	DON + FBs + OTA (%)	DON + FBs + ZEN (%)
Feed ingredients	Grain	12	2 (16.7)	-	-	-	-	1 (8.3)	-	1 (8.3)
	Bran	25	12 (48.0)	1 (4.0)	-	-	-	5 (20.0)	-	6 (24.0)
	Animal protein	27	-	-	-	-	-	-	-	-
	Vegetable meal	40	10 (25.0)	-	-	-	1 (2.5)	3 (7.5)	-	6 (15.0)
	Mineral	13	-	-	-	-	-	-	-	-
	Mix	63	6 (9.5)	1 (1.6)	-	-	-	3 (4.8)	-	2 (3.2)
Compound feeds	Poultry	38	31 (81.6)	5 (13.2)	-	-	-	3 (7.9)	1 (2.6)	22 (57.9)
	Pig	53	42 (79.2)	6 (11.3)	-	-	-	3 (5.7)	-	33 (62.3)
	Dairy	20	19 (95.0)	1 (5.3)	-	-	-	9 (45.0)	-	9 (45.0)
	Cattle	42	40 (95.2)	3 (7.1)	-	-	-	13 (31.0)	-	24 (57.1)
	Pet	403	160 (39.7)	35 (8.7)	3 (0.7)	1 (0.2)	-	57 (14.1)	-	64 (15.9)

^1^ DON, deoxynivalenol; ^2^ FBs, fumonisin B_1_ and B_2_; ^3^ ZEN, zearalenone; ^4^ OTA, ochratoxin A.

**Table 3:** Optimized multiple-reaction monitoring (MRM) parameters for quantification and qualification of the 11 mycotoxins and internal standards (ISs).

Compound	Ionization	RT (min)	Precursor Ion (*m*/*z*)	Quantitative Ion (*m*/*z*)	Qualitative Ion (*m*/*z*)	Collision Energy (eV)
Aflatoxin B_1_	[M+H]^+^	10.7	313.0	285.0	241.0	−25, −40
Aflatoxin B_2_	[M+H]^+^	10.4	315.0	259.1	287.2	−30, −27
Aflatoxin G_1_	[M+H]^+^	10.2	328.9	243.1	311.1	−27, −23
Aflatoxin G_2_	[M+H]^+^	9.7	331.0	313.1	245.2	−26, −31
Deoxynivalenol	[M+H]^+^	6.4	297.0	249.3	231.1	−13, −14
Fumonisin B_1_	[M+H]^+^	11.9	722.3	352.2	334.3	−37, −40
Fumonisin B_2_	[M+H]^+^	13.1	706.3	336.4	354.2	−38, −33
Ochratoxin A	[M+H]^+^	13.2	403.9	239.1	358.1	−25, −16
T-2 toxin	[M+NH_4_]^+^	12.6	484.2	215.2	305.2	−21, −16
HT-2 toxin	[M+NH_4_]^+^	11.9	442.0	263.2	215.2	−15, −15
Zearalenone	[M−H]^−^	13.3	317.0	175.3	131.3	24, 29
^13^C_17_ Aflatoxin B_1_ (IS)	[M+H]^+^	10.6	330.0	301.1	255.1	−25, −38
^13^C_17_ Aflatoxin B_2_ (IS)	[M+H]^+^	10.3	332.0	303.1	257.1	−28, −40
^13^C_17_ Aflatoxin G_1_ (IS)	[M+H]^+^	9.9	346.0	257.0	328.1	−29, −24
^13^C_17_ Aflatoxin G_2_ (IS)	[M+H]^+^	9.6	348.0	330.1	301.2	−25, −29
^13^C_15_ Deoxynivalenol (IS)	[M+H]^+^	6.4	312.0	215.1	198.4	−22, −21
^13^C_34_ Fumonisin B_1_ (IS)	[M+H]^+^	11.8	756.0	374.4	356.4	−39, −42
^13^C_34_ Fumonisin B_2_ (IS)	[M+H]^+^	13.1	740.0	376.4	358.4	−36, −39
Ochratoxin A-d5 (IS)	[M+H]^+^	13.2	409.2	363.2	239.1	−17, −25
^13^C_24_ T-2 toxin (IS)	[M+NH_4_]^+^	12.5	508.4	322.2	229.2	−16, −20
^13^C_22_ HT-2 toxin (IS)	[M+NH_4_]^+^	11.8	464.0	278.2	229.2	−16, −16
^13^C_18_ Zearalenone (IS)	[M−H]^−^	13.2	335.0	185.2	140.2	25, 31
